# Representation of Psoriasis on the Web for Patients With Skin of Color

**DOI:** 10.2196/69026

**Published:** 2025-08-05

**Authors:** Daniel Nguyen, Van Le, Derek Nguyen, Vy Han

**Affiliations:** 1 California University of Science and Medicine, Colton, CA, United States; 2 University of California, Riverside, 900 University Ave, Riverside, CA, 92521, United States, 1 7148802250

**Keywords:** Fitzpatrick skin type, Instagram, social media representation, psoriasis, internet

## Abstract

This study analyzed over 2000 images of psoriasis across major web-based platforms and found a significant underrepresentation of darker skin tones, highlighting a critical gap in dermatologic representation that may contribute to misdiagnoses and health disparities among patients with skin of color.

## Introduction

Over recent decades, the internet has grown in popularity as a primary health information source, with 74.4% of US adults reporting that they consult it before turning to other resources [1]. Among web-based platforms, social media has emerged as a widely used educational tool for accessing health-related information [2]. Psoriasis—a lifelong inflammatory skin disease affecting around 125 million people worldwide [3]—is theorized to be underdiagnosed among patients with skin of color (SOC), possibly due to lack of access to health care and nuances in the disease’s manifestation [4]. In patients with darker skin tones, psoriatic lesions may appear grayish or violaceous rather than the typical salmon pink, leading to misdiagnosis as postinflammatory hyperpigmentation and contributing to disease persistence and undertreatment [5]. For early detection, medical education, and public awareness, SOC representation is important in images of psoriasis on the web. This study assesses skin tone diversity in depictions of psoriasis on Google Images, Instagram (Meta Platforms), Facebook (Meta Platforms), YouTube (Google LLC), and DermNet, using the Fitzpatrick scale.

## Methods

On April 13 and 14, 2025, we performed searches for “psoriasis” on Google Images, Instagram, YouTube, Facebook, and DermNet, as these represented the most popular sources of consumer health information, particularly among people of color; Facebook, YouTube, and Instagram each show usage rates exceeding 50% in this demographic [6]. In total, over 2000 images depicting patients with psoriasis were retrieved from these platforms. Computer-generated images, duplicate images on the same platform, images with poor lighting, and images featuring the same patient at a different angle were excluded from data collection. On YouTube, 500 images of individual patients with psoriasis were extracted from 163 videos. To minimize algorithmic bias, searches were performed by using incognito browsers, a new social media account, and 3 different IP addresses. Extracted images were independently categorized based on skin tone by 3 reviewers using the Fitzpatrick scale. Disagreements on classification were resolved by majority vote. Images were further designated as light skin images (Fitzpatrick skin types I, II, III, and IV) or dark skin images (Fitzpatrick skin types V and VI) [7]. The quantities of dark skin and light skin images were compared using a 2-tailed *t* test. A *P* value of <.05 was considered statistically significant.

## Results

Images of psoriasis (n=2341) in Fitzpatrick type II skin were the most abundant across all platforms, with 56.4% (1320/2341) of images constituting that classification (Table 1). Interrater reliability was substantial (Cohen κ=0.76). Dark skin images of psoriasis and images of the lightest skin tone—Fitzpatrick type I—were relatively few on all 5 platforms. In total, 5.2% (122/2341) of psoriasis images were dark skin images, and 94.8% (2219/2341) were light skin images, representing a significant difference (*P*<.001). Notably, Fitzpatrick type IV skin had low representation on Google Images (27/401, 6.8%) and YouTube (21/500, 4.2%) and higher representation on Instagram (52/500, 10.4%).

**Table 1.** Representation of different skin types in photos of psoriasis on Google Images, Instagram, Facebook, YouTube, and DermNet.

Internet resources	Total, n	Fitzpatrick skin type, n (%)						Dark, n (%)	Light, n (%)
		Type I	Type II	Type III	Type IV	Type V	Type VI		
Google Images	401	35 (8.7)	219 (54.6)	100 (24.9)	27 (6.8)	11 (2.8)	9 (2.2)	20 (5.0)	381 (95.0)
Instagram	500	49 (9.8)	266 (53.2)	107 (21.4)	52 (10.4)	19 (3.8)	7 (1.4)	26 (5.2)	474 (94.8)
Facebook	500	26 (5.2)	300 (60.0)	96 (19.2)	40 (8)	24 (4.8)	14 (2.8)	38 (7.6)	462 (92.4)
YouTube	500	34 (6.8)	294 (58.8)	127 (25.4)	21 (4.2)	9 (1.8)	15 (3.0)	24 (4.8)	476 (95.2)
DermNet	440	36 (8.2)	241 (54.8)	107 (24.3)	42 (9.5)	7 (1.6)	7 (1.6)	14 (3.2)	426 (96.8)

## Discussion

Our findings suggest that social media postings of patients with psoriasis and darker skin types are underrepresented across all platforms. These results align with research examining SOC representation within medical education, indicating this issue’s prevalence across many information sources [8]. Furthermore, the underrepresentation extends to psoriasis-related content in dermatology residency teaching materials, wherein patients with SOC may also be inadequately depicted [4]. These oversights contribute to the underdiagnosis of psoriasis and poorer outcomes for individuals with SOC, as the disease’s clinical presentation can differ across racial and ethnic groups. Given that patients may access the internet for information before visiting a dermatologist, greater image diversity would also be helpful to patients with SOC who suspect that they have psoriasis. Barriers to improving representation on the web include cultural perceptions of psoriasis, which can influence health care–seeking behavior in certain racial and ethnic groups [4]. For example, psoriasis-related stigma is particularly prevalent among Black and Latino patients, further discouraging them from sharing their images and experiences on the web [4,9]. Our study’s limitations include potential hyperpigmentary effects influencing raters’ judgments and algorithmic bias in Google search results, which may vary by geographic region, despite the use of 3 different IP addresses. Our results demonstrate that internet companies have cause to improve psoriasis representation in search results. The limited content available for people of color highlights a gap that content creators, health care professionals, and social media platforms must address to improve representation and reduce health disparities in psoriasis care. Future efforts should be directed toward improving the quality and dissemination of photographs of psoriasis in SOC.

## Abbreviations

SOC: skin of color
